# Low PEEP Mechanical Ventilation and PaO_2_/FiO_2_ Ratio Evolution in COVID-19 Patients

**DOI:** 10.1007/s42399-021-01031-x

**Published:** 2021-07-24

**Authors:** Samuele Ceruti, Marco Roncador, Andrea Saporito, Maira Biggiogero, Andrea Glotta, Pier Andrea Maida, Patrizia Urso, Giovanni Bona, Christian Garzoni, Romano Mauri, Alain Borgeat

**Affiliations:** 1 Department of Critical Care, Clinica Luganese Moncucco, Lugano, Switzerland; 2 Internal Medicine Service, Clinica Luganese Moncucco, Lugano, Switzerland; 3 Department of Medical Oncology and Hematology, University Hospital Zürich, Zürich, Switzerland; 4 Anesthesiology Division, Ospedale Regionale di Bellinzona e Valli, 6500 Bellinzona, Switzerland; 5 Clinical Research Unit, Clinica Luganese Moncucco, Lugano, Switzerland; 6 Radiotherapy Department, Clinica Luganese Moncucco, Lugano, Switzerland; 7 Anaesthesiology Division, Balgrist University Hospital, Zürich, Switzerland

**Keywords:** Adult respiratory distress syndrome, COVID-19, Mechanical ventilation, PaO_2_/FiO_2_, Positive-end-expiratory respiration

## Abstract

Invasive mechanical ventilation (IMV) is the standard treatment in critically ill COVID-19 patients with acute severe respiratory distress syndrome (ARDS). When IMV setting is extremely aggressive, especially through the application of high positive-end-expiratory respiration (PEEP) values, lung damage can occur. Until today, in COVID-19 patients, two types of ARDS were identified (L- and H-type); for the L-type, a *lower PEEP strategy* was supposed to be preferred, but data are still missing. The aim of this study was to evaluate if a clinical management with lower PEEP values in critically ill L-type COVID-19 patients was safe and efficient in comparison to usual standard of care. A retrospective analysis was conducted on consecutive patients with COVID-19 ARDS admitted to the ICU and treated with IMV. Patients were treated with a *lower PEEP strategy* adapted to BMI: PEEP 10 cmH_2_O if BMI < 30 kg m^−2^, PEEP 12 cmH_2_O if BMI 30–50 kg m^−2^, PEEP 15 cmH_2_O if BMI > 50 kg m^−2^. Primary endpoint was the PaO_2_/FiO_2_ ratio evolution during the first 3 IMV days; secondary endpoints were to analyze ICU length of stay (LOS) and IMV length. From March 2 to January 15, 2021, 79 patients underwent IMV. Average applied PEEP was 11 ± 2.9 cmH_2_O for BMI < 30 kg m^−2^ and 16 ± 3.18 cmH_2_O for BMI > 30 kg m^−2^. During the first 24 h of IMV, patients’ PaO_2_/FiO_2_ ratio presented an improvement (*p*<0.001; CI 99%) that continued daily up to 72 h (*p*<0.001; CI 99%). Median ICU LOS was 15 days (10–28); median duration of IMV was 12 days (8–26). The ICU mortality rate was 31.6%. Lower PEEP strategy treatment in L-type COVID-19 ARDS resulted in a PaO_2_/FiO_2_ ratio persistent daily improvement during the first 72 h of IMV. A *lower PEEP strategy* could be beneficial in the first phase of ARDS in critically ill COVID-19 patients.

## Background

Severe acute respiratory syndrome coronavirus 2 (SARS-CoV-2) is the cause of COVID-19, a pandemic that has affected more than 180,000,000 individuals and caused nearly 4,000,000 deaths since initial detection of the virus at the end of January 2019 [1]. Invasive mechanical ventilation (IMV) is the gold standard treatment in critically ill COVID-19 patients with acute respiratory distress syndrome (ARDS); in this scenario, ventilatory settings with increased positive-end-expiratory respiration (PEEP) values have been suggested [2–4]. However, when IMV setting is extremely aggressive, as in the case of high PEEP values, pulmonary complications like barotrauma, volutrauma, or biotrauma can occur [5–7].

Relevant pathophysiological understanding of COVID-19 reported by Cronin [8], Nieman [9], Gattinoni [10], and Bendjelid [11] identified specific lung features in the early stages of the disease. According to these features, Habashi et al. stratified COVID-19 ARDS in two different groups, identifying a L-type and a H-type ARDS [12] based on different lung pathophysiology. The first population presents a higher lung compliance compared to “classic” ARDS patients, due to a probable alveolitis, with a shunt effect due to loss of local hypoxic vasoconstriction. The second population described presents a low lung compliance and a pattern of “baby-lung” compatible with classic ARDS [2, 3, 10, 11, 13–15]. More recent evidences suggested that in patients with L-type ARDS a less aggressive approach implementing a *lower PEEP strategy* may be favorable [2, 16–19]. PEEP values between 8 and 10 cmH_2_O, intended as *lower PEEP strategy*, were suggested to be adequate in this setting [18].

During ARDS, also in the setting of critically ill COVID-19 patients, the PaO_2_/FiO_2_ ratio is typically used as a prognostic stratification parameter [20]; moreover, it determines lung respiratory efficiency, acting as a primary clinical indicator of hypoxemia [20], allowing to properly evaluate changes in patients’ respiratory status. The aim of this project was to verify if, in critically ill COVID-19 patients, a clinical management implementing a *lower PEEP strategy* during IMV was safe and efficient comparing to usual standard of care, analyzing the PaO_2_/FiO_2_ ratio.

## Methods

After approval by the Ethical Committee (Ethics Committees of Canton Ticino; Dec 2020, CE TI 3775) and in accordance with local federal rules, a retrospective analysis was conducted on consecutive patients with acute respiratory distress due to COVID-19 pneumonia admitted to the ICU during two pandemic waves (from March 2 to April 10, 2020, and from October 5, 2020, to January 15, 2021). All critically ill COVID-19 patients’ relevant data like demographics, severity score (NEMS — nine equivalents of nursing manpower use score, SAPS — simplified acute physiology score), clinical information, and laboratory/radiological results were obtained during patients’ hospitalization from electronic health records. Standard laboratory tests included complete blood count, CRP, ferritin, ASAT, ALAT, blood ionogram, creatinine, urea, D-dimer, Prothrombin Time (PT), activated partial thromboplastin time (aPTT), fibrinogen, blood gas analysis, SvO_2_, NT-pro-BNP, blood, and urine cultures and urine analysis for *Legionella pneumophila* antigen. All patients underwent chest x-ray and transthoracic echocardiography, to assess the global cardiac function before every pronation cycle. Performing a chest CT scan was considered at ICU admission, if the examination had not been performed within the preceding 24 h.

At ICU admission, COVID-19 patients underwent non-invasive ventilation (NIV) through C-PAP or high-flow nasal cannula (HFNC); in case of worsening, defined as 2 consecutive ROX-index lower than 3.85 [21, 22] or residual respiratory distress after 1 h of NIV, patients underwent IMV. Patients who did not develop neither dyspnea nor worsening in ROX-index were treated with NIV.

After endotracheal intubation, a *lower PEEP strategy* based on BMI was adopted: PEEP values of 10, 12, and 15 cmH_2_O were applied, respectively, for patients with BMI <30, 30–50, and >50 kg/m^2^. Once PEEP adjustment was performed, according to the *ARDSnet PEEP table* [13, 23] and PV-tools ventilatory measurements, FiO_2_ was adapted to maintain a SpO_2_ greater than 92% and a PaO_2_ > 60 mmHg (8 kPa). A protective ventilation strategy (TV 6–8 ml kg^−1^, *P*_plat_ < 30 cmH_2_O) with permissive hypercapnia (pH > 7.20) was adopted [24], with pronation cycles of 16 h beginning at the admission. A deep sedation was maintained to pursue a Richmond Agitation and Sedation Scale (RASS) of −4 during the first 36 h, combined with muscle relaxation in case of patient-ventilator asynchrony [25]. Given the thrombogenic diathesis of COVID-19 patients [24, 26], all patients were treated with an intermediate-prophylaxis, switched to a therapeutic dose in case of high risk of venous thromboembolism [27]. All clinical, ventilatory, and biological data were reported.

Primary endpoint was to report the PaO_2_/FiO_2_ ratio evolution during IMV after application of lower PEEP according to BMI during the first 3 days of IMV. Secondary endpoints were to report and analyze patients ICU LOS and IMV length, further describing patients’ demographic characteristics, clinical complications rate, and critical care outcome.

### Statistical Analysis

Descriptive statistics was performed to summarize the collected clinical data. Gaussian distribution was verified by Kolmogorov-Smirnov test. Differences between patient outcomes were studied with a *t*-test for independent groups or with a Mann-Whitney test if a non-parametric analysis was required. Similarly, comparison of clinical evolution over time was performed with a paired *t*-test or with a non-parametric Wilcoxon test, depending on data distribution. A study of differences between groups of categorical data was carried out with chi-square statistics. The significance level of *p* value was established to be <0.01, with a confidence interval (CI) of 99%. Statistical data analysis was performed using the SPSS.26 package (SPSS Inc., Armonk, NY; USA).

## Results

### ICU Patients’ Characteristics

**Fig. 1:**
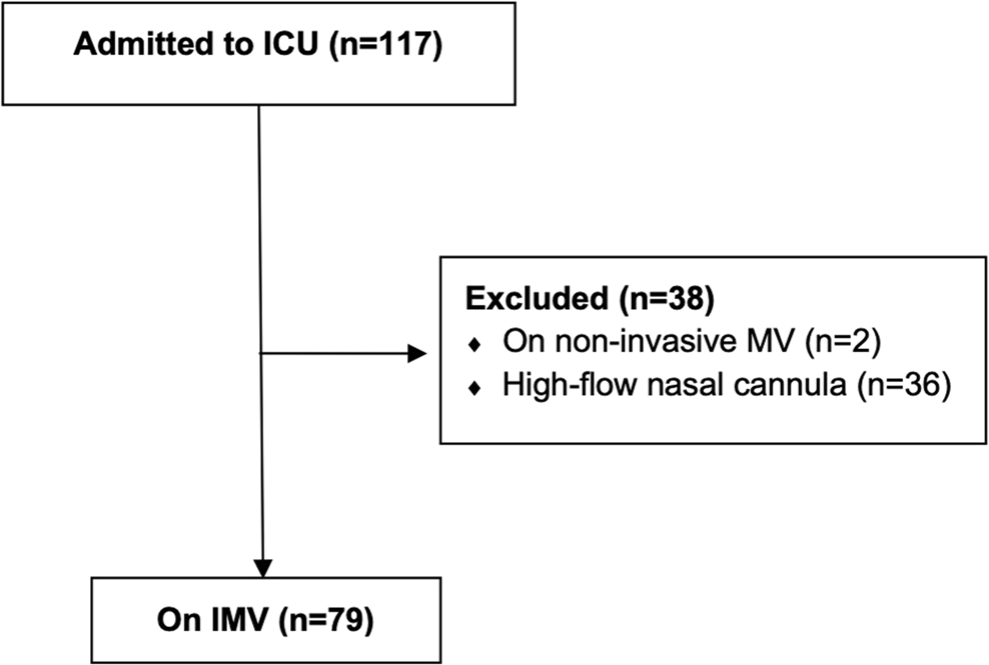
CLM COVID-19 patients. Management of COVID-19 patients evaluated at our COVID-19 center during two pandemic waves (from March 2nd to April 10th, 2020, and from October 5th, 2020, to January 15th, 2021). ICU admission was performed according to standard selection criteria (SpO_2_ < 85% and/or dyspnea and/or signs of mental confusion). Patients not on invasive MV were excluded from the analysis.

During the first pandemic wave, 46 patients were admitted to the ICU, while during the second wave 71 patients were further admitted. Thirty-eight patients did not receive IMV and were therefore excluded from the analysis; the seventy-nine patients who received IMV were instead included in the study (Fig. 1). Mean age was 67 ± 11 years; most patients were men (81%), often with one or more chronic medical conditions, most commonly arterial hypertension (57%) and diabetes (35.7%); almost all patients resulted hemodynamically stable (95%), without needing of inotropic agent (Table 1). A chest CT scan was obtained in 64 (76.2%) patients, showing bilateral ground-glass opacities in all cases, as well as concomitant consolidations in 13 of them (15.5%). Regarding the physiology of the patients’ lungs, the mean lung compliance resulted 63 ml/cmH_2_O (SD 6 ml/cmH_2_O), with mean pCO_2_ of 43.9 mmHg (SD 9.1 mmHg); FiO_2_ level was reduced comparing the pre-/post-intubation phases, respectively, from a median of 95% (80–100) to a median of 70% (60–90). Demographic and clinical data are reported in Table 1; basal respiratory data are reported in Table 2.

**Table 1:** Baseline characteristics

	Unit	n.v.	Results
Demographic data			
Patients on invasive MV	n		79
Age	Years		67 ± 11 (29–86)
Male	n		68 (81%)
BMI	kg m^−2^		29 ± 5.1 (18.6–44.9)
SAPS			47 ± 17 (13–94)
NEMS			34 ± 9 (9–49)
Comorbidities			
Arterial hypertension	n		48 (57.1%)
Ischemic cardiopathy	n		19 (22.6%)
Diabetes	n		30 (35.7%)
Obstructive sleep apnea syndrome	n		9 (10.7%)
COPD	n		12 (14.3%)
Mean duration of symptoms	Days		5 (1–29)
At admission			
Hemodynamics			
Systolic arterial pressure	mmHg	110–140	129 (120–140)
Diastolic arterial pressure	mmHg	60–80	65 (60–75)
Heart rate	bpm	60–100	85 (50–96)
Temperature	°C	36–38.3	37.0 (36.3–37.6)
Lactate	mmol L^−1^	< 2.0	1.2 (0.8–1.6)
Inotropic drug	n		0
Laboratory			
ASAT	U L^−1^	10–50	47 (36–72)
ALAT	U L^−1^	10–50	33 (21–48)
Leucocyte	G L^−1^	4.0–10.0	6.8 (4.8–10)
Lymphocyte	G L^−1^	1.3–3.6	0.7 (0.5–1.0)
Radiology			
Chest x-ray	n		61 (72.6%)
Chest CT scan			
- No CT scan	n		20 (23.8%)
- Ground glass	n		51 (60.7%)
- Ground glass and consolidation	n		13 (15.5%)

Demographic characteristics and blood tests at ICU admission. Continuous measurements were presented as mean ± SD (min–max) otherwise as median (25th–75th) if they are not normally distributed. Categorical variables were reported as counts and percentages

**Table 2:** MV setting and clinical outcomes

	Unit	n.v.	Results
Basal respiratory data			
FiO_2_ at admission before OTI	%		95 (80–100)
P/F ratio at admission before OTI		> 300	70 (54–101)
FiO_2_ after OTI	%		70 (60–90)
P/F ratio after OTI		> 300	145 (111–206)
Ventilatory strategy			
PEEP-strategy			
- BMI < 30 kg/m^2^	cmH_2_O		11 ± 2.9 (10–12)
- BMI 30–50 kg/m^2^	cmH_2_O		16 ± 3.18 (12–18)
- BMI > 50 kg/m^2^	cmH_2_O		NA
Respiratory data evolution			
Lung dynamic compliance	ml/cmH_2_O	50–100	63 ± 6
pCO_2_	mmHg	35–45	43.9 ± 9.1
P/F ratio at first day		> 300	120 (94–174)
P/F ratio at second day		> 300	160 (120–220)
P/F ratio at third day		> 300	197 (140–235)
Pronation cycles	n		4 (2–5)
Laboratory data (first 72 h)			
C-reactive-protein max	mg L^−1^	< 5	185 (106–258)
Ferritin max	ng mL^−1^	30–500	1819 (878–3200)
Lactate de-hydrogenase max	U L^−1^	135–225	530 (402–695)
Creatinine max	umol L^−1^	62–106	93 (72–129)
Troponin T hs max	ng L^−1^		14.8 (8.1–43.0)
Creatinine kinase max	U L^−1^	39–308	235 (101–360)
Platelets min	G L^−1^	150–450	171 (133–236)
Bilirubin total max	μmol L^−1^	< 21.0	8.3 (6.4–12.5)
Clinical outcome			
Length of ICU stay	Days		15 (10–28)
Length of MV	Days		12 (8–26)
Complications			
Thromboembolism confirmed	n		19 (24.1%)
Massive hemorrhage	n		6 (7.6%)
VAP	n		37 (46.8%)
AKI needing RRT	n		10 (11.9%)

ICU respiratory data at admission, during treatment and PEEP strategy, with ICU MV and laboratory data. Continuous measurements were presented as mean ±SD (min–max) otherwise as median (25th–75th) if they are not normally distributed. Categorical variables were reported as counts and percentages

### IMV Settings with Low PEEP

**Fig. 2:**
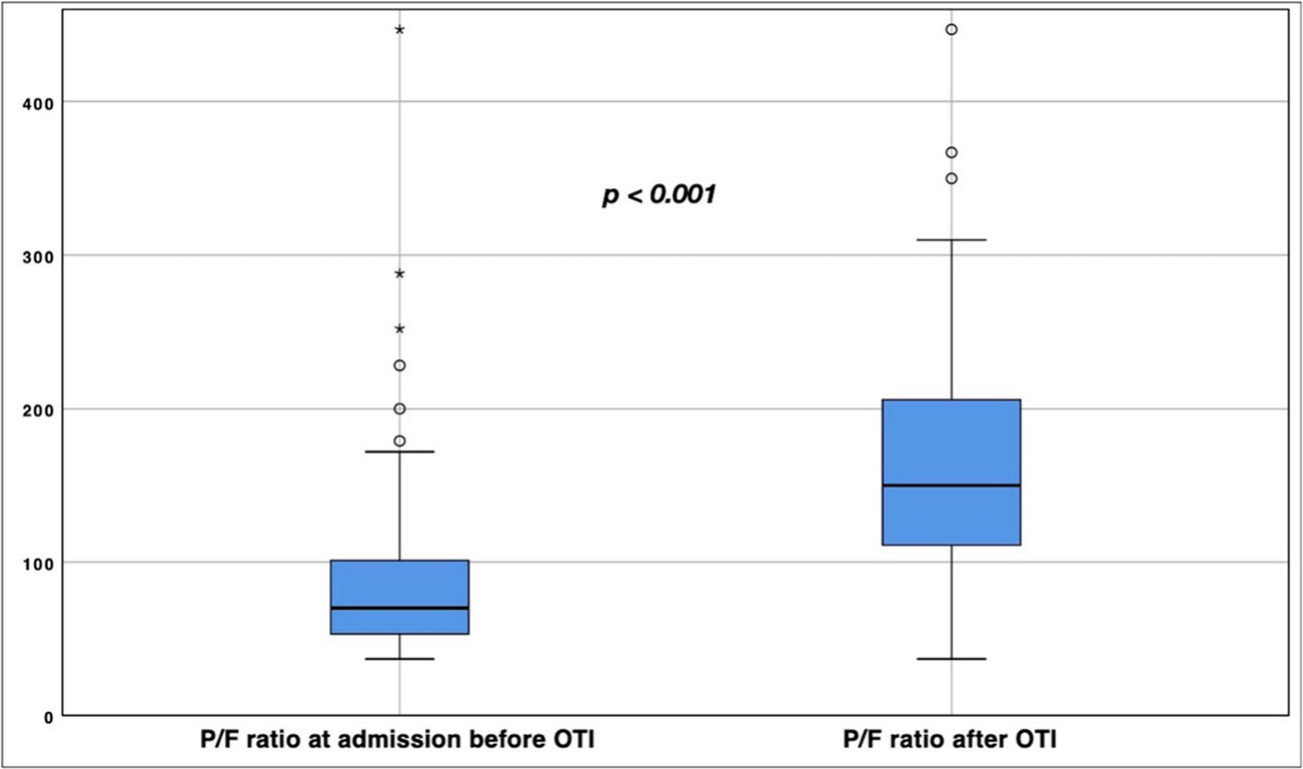
P/F ratio variation at OTI. P/F ratio variation before/after OTI at ICU admission (−75, CI 99%, −98/−52, *p* < 0.001)

For patients with BMI < 30 kg m^−2^, mean titrated PEEP was 11 cmH_2_O (SD 2.9), while for patients with BMI > 30 kg m^−2^ the mean PEEP was 16 cmH_2_O (SD 3.18); no patients with BMI more than 50 kg m^−2^ were admitted in the ICU. Upon ICU admission, early P/F ratio reported a median of 70 (54–101), with a median FiO_2_ of 95% (80–100) with non-invasive medical oxygen supply. After the implementation of IMV, the median FiO_2_ resulted 70% (60–90), with a median first P/F ratio of 145 (111–206), significantly increased compared to the P/F ratio preceding IMV (−75, CI 99%, −97/−52, *p* < 0.001) (Fig. 2). Seventy-eight (92.8%) patients underwent pronation cycles, with a median of 4 cycles per patient (Table 2).

**Fig. 3:**
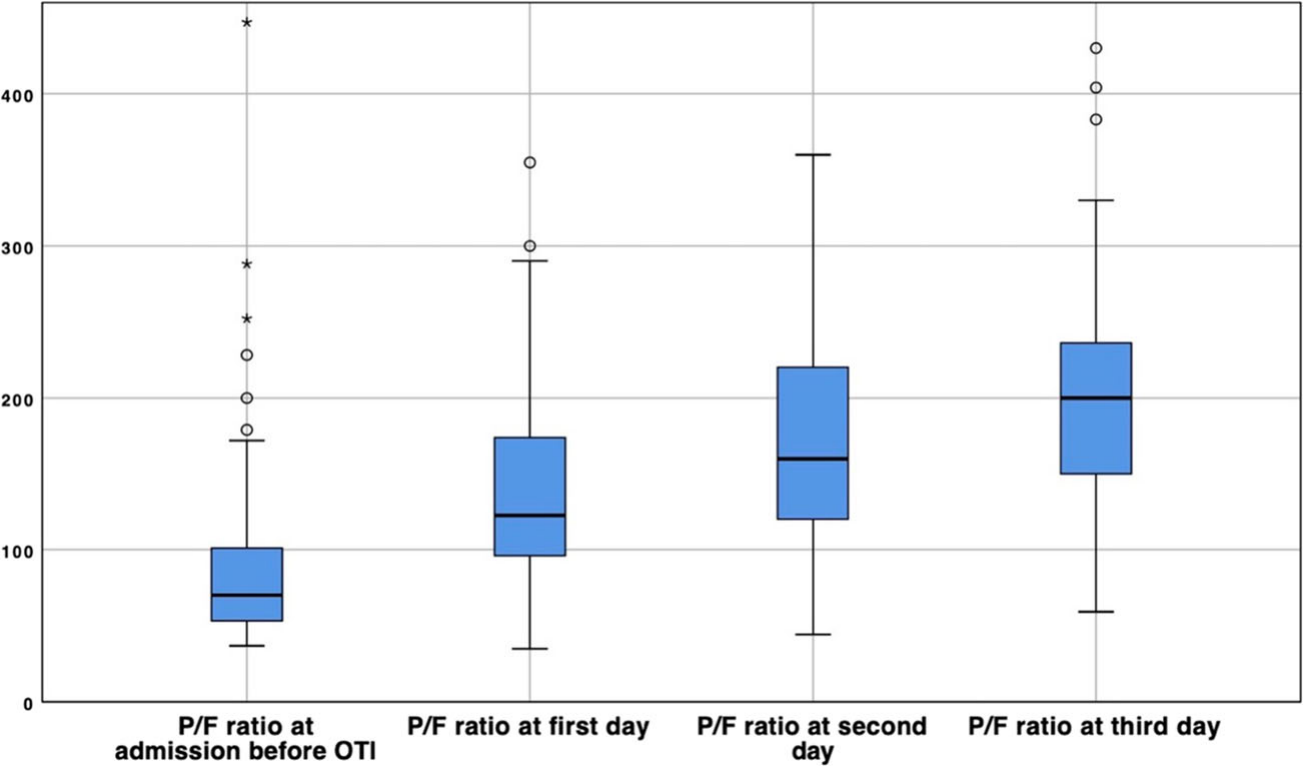
P/F ratio variation during MV. P/F ratio variation at ICU admission compared to the first, second, and third day of MV. All daily median PF values resulted significantly different compared to admission and compared to the day after, even with the use of low PEEP setting on MV. All differences resulted statistically significative (CI 99%, *p* < 0.001)

The P/F ratio after low PEEP application (Table 2) progressively improved, with a median value of 120 (94–174) at day 1 (−44, CI 99%, −64/−24, *p* < 0.001), 160 (120–220) on the second day (−81, CI 99%, −103/−60, *p* < 0.001), and 197 (140–235) on day 3 (−106, CI 99%, −133/−78, *p* < 0.001). This improvement resulted statistically significant both when comparing the P/F ratio between the first and second day (−36, CI 99%, −50/−22, *p*<0.001) and between the second and third day (−23, CI 99%, −40/−6, *p*<0.001) (Fig. 3).

### ICU Patient Outcome

Median ICU LOS was 15 days (10–28); the median duration of IMV length resulted 12 days (8–26) (Table 2). At 28 days, 41 critically ill patients (48.8%) were discharged from the ICU, 8 inpatients (9.5%) were still receiving IMV (6 via endotracheal tube, 2 via tracheostomy), and 5 patients (4.8%) were transferred to another hospital. The ICU mortality rate was 31.6% (25 patients). No patient underwent reintubation within or after 72 h from extubation. After the analysis of patients’ survival, no specific variable was significantly associated with better survival, both at clinical level, like age (mean 72.2 vs 64.5 years, *p*=0.056), and at biological level, like serum leucocyte (median 7 vs 8 G/L, *p*=0.09), lymphocyte (median 0.6 vs 0.9 G/L, *p*=0.08), and CK values (median 198 vs 232 U/L, *p*=0.91).

### Clinical Complications

Nineteen patients (24.1%) presented major VTE phenomena (16 pulmonary embolism, 3 veno-arterial thrombosis) and 8 patients (9.5%) presented deep vein thrombosis. Fifteen (17.8%) patients received anticoagulation at a prophylactic dose, while 60 (71.4%) patients received a full therapeutic dosage. No patient presented any contraindication to parenteral anticoagulation; 6 (7.6%) patients presented bleeding complication, requiring anticoagulation suspension and specialist treatment. Thirty-seven (46.8%) patients undergoing IMV were diagnosed with ventilator associated pneumonia (VAP) and subsequently treated with antibiotic therapy in accordance with local clinical practice. Nineteen (24.1%) patients presented acute kidney injury (AKI), with 10 (11.9%) patients requiring renal replacement therapy (RRT) implementation.

## Discussion

Acute respiratory distress induced by SARS-CoV-2 is a critical clinical condition associated with COVID-19 infection [28, 29]. In a multisystem disease such as COVID-19, a multidisciplinary approach is recommendable [30]; to minimize the high mortality rate potentially associated with COVID-19 pandemics, and to correctly manage this critical condition, adequate hospital resources, structured triage, and appropriate clinical training are required [30]. Even if the classic ARDS criteria were identified in COVID-19 patients [31], clinical evidences led us to consider that atypical aspects were also evident, especially the lack of a reduced lung compliance with consequent hypercapnia [32]. The lack of “baby-lung” pattern [33] and the peculiar physiological characteristics of this ARDS [15, 17, 19, 31], suggested the implementation of a specific ventilation setting, in particular concerning PEEP [18]. Based on the abovementioned aspects, we specifically applied a *lower PEEP* ventilatory setting to all patients receiving IMV, carefully tailored to lungs physiology and, in agreement to *ARDSnet PEEP table*, also to BMI [17, 21, 28, 32]. As expected, after endotracheal intubation, we found patients easy to ventilate, with an average lung compliance higher than classic ARDS, without any sign of pCO_2_ retention [34].

This approach was consistent with growing evidences, as suggested by Gattinoni et al. [33] and Bendjelid et al. [11], who identified the presence of two different ICU patient populations in COVID-19 ARDS. Although our strategy was different compared to the available literature [35] and to recent NIH guidelines [4], there are an ever-increasing number of evidences suggesting a *lower PEEP strategy* in the management of L-type ARDS, based on specific lung physiology [15, 16, 18]. In these cases, higher PEEP could cause lung overdistension, resulting in an increased driving pressure and lung damage [36–40]; moreover, PEEP levels greater than 10 cmH_2_O can induce the reduction of venous return, with consequent worsening of the circulation status, as well as local biotrauma and a worsening of alveolar damage [17, 37, 38]. In fact, a *lower PEEP* approach resulted beneficial in our patients’ cohort; ventilatory data confirmed a rapid improvement in the oxygenation lung function already in the first 3 days after endotracheal intubation, without any sign of hypoventilation, suggesting that L-type ARDS [3, 10, 14, 15] appeared to respond appropriately to *lower PEEP* treatment tailored to patients’ BMI. Moreover, this ventilatory setting avoided us to induce both a reduction in alveolar ventilation and a worsening in arterial oxygen saturation due to alveolar over/under-distension.

Other groups reported a 50% and a 61.5% of death rate [41]; in our critically ill COVID-19 patients cohort, we registered a mortality rate of 31.6%. We supposed that a relatively low-pressure ventilation could prevent the transition from an initial alveolitis to an iatrogenic H-type ARDS, in which the ongoing inflammation is worsened by high levels of PEEP, through a ventilation-induced-lung injury (VILI) mechanism. Moreover, our patients cohort median ICU LOS was reported to be equivalent to other groups, like Bhatraju et al. [41], even including patients who were deceased in the ICU. This data could suggest that a *lower PEEP strategy* with a protective IMV approach can improve COVID-19 patients’ in-hospital management, morbidity and mortality, although further studies are necessary to confirm this interesting hypothesis.

Our project was burdened by several limitations. Firstly, this study compared lower PEEP in consecutive critically ill COVID-19 patients, and it was not possible to compare the data to a control group, even if the reproducible results obtained in the two distinct pandemic waves strongly support our analysis. Secondly, it was a monocentric observational retrospective study, with a relatively small series of patients. In this regard, a comparison with current literature was performed; even if patient populations differed, results can be assumed to be consistent, as the cohorts are comparable in terms of disease severity and biochemical markers.

## Conclusion

A *lower PEEP treatment* in critically ill COVID-19 patients on IMV resulted in rapid and progressive improvement of PaO_2_/FiO_2_ ratio during the first 72 h. A more physiology-based IMV setting could help to implement the understanding of the ARDS pathophysiological mechanisms in COVID-19 patients management; further studies need to be performed to confirm this approach.

## Data Availability

The datasets used and/or analyzed during the current study are available from the corresponding author on reasonable request.
